# Modification of Commercial Polymer Coatings for Superhydrophobic Applications

**DOI:** 10.1021/acsomega.3c09123

**Published:** 2024-02-02

**Authors:** Sam S. Cassidy, Kristopher Page, Cesar III De Leon Reyes, Elaine Allan, Ivan P. Parkin, Claire J. Carmalt

**Affiliations:** † Materials Chemistry Research Centre, Department of Chemistry, University College London, London WC1H 0AJ, U.K.; ‡ Department of Microbial Diseases, UCL Eastman Dental Institute, Royal Free Campus, University College London, London WC1H 0AJ, U.K.

## Abstract

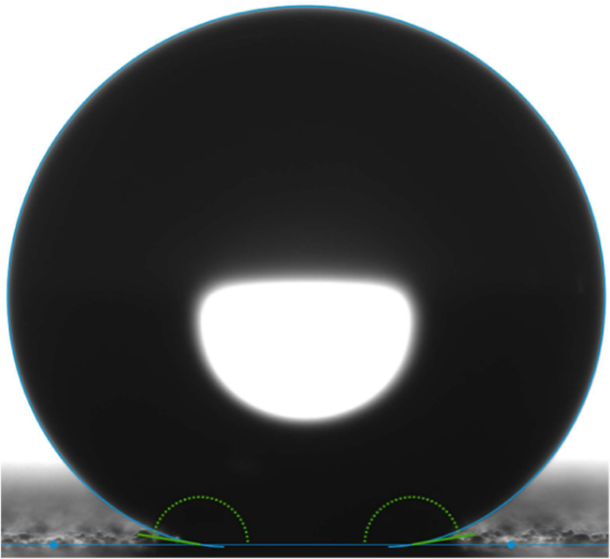

Superhydrophobic surfaces have been studied extensively over the past 25 years. However, many industries interested in the application of hydrophobic properties are yet to find a suitable solution to their needs. This paper looks at the rapid functionalization of nanoparticles and the fabrication of superhydrophobic surfaces with contact angles > 170°. This was achieved by simply mixing commercial products and applying the new formulation with scalable techniques. First, inexpensive and nontoxic superhydrophobic nanoparticles were made by functionalizing nanoparticles with fatty acids in under an hour. A similar methodology was then used to functionalize a commercial polymer coating to express superhydrophobic properties on it by lowering the coating’s surface energy. The coating was then applied to a surface by the spray technique to allow for the formation of hierarchical surface structures. By combining the low surface energy with the necessary roughness, the surface was able to express superhydrophobic properties. Both the particles and the surfaces then underwent characterization and functional testing, which, among other things, allowed for clear differentiation between the functionalization properties of the zinc oxide (ZnO) and the silica (SiO_2_) nanoparticles. This paper shows that suitable superhydrophobic solutions may be found by simple additions to already optimized commercial products.

## 1 Introduction

Superhydrophobic surfaces, a surface with a water contact angle (WCA) > 150°, may have applications as water repellent surfaces, self-cleaning surfaces, anti-icing surfaces, antibiofouling, etc.^1−3^ Since the 1950s, multiple drug-resistant pathogens have begun to emerge, predominantly due to our over-reliance on antibiotics.^4^ A continued arms race between antibiotics and pathogens led the World Health Organization to declare drug-resistant pathogens as one of the top global public health threats facing humanity.^5^ Superhydrophobic surfaces have been shown to reduce the spread of pathogens through the prevention of colonization of the surface, reducing the need for antibiotic use.^6^ While these properties are desired by the textile and health industries for obvious reasons, self-cleaning and anti-icing properties are also required by the energy industry. As we continue to move away from fossil fuels, the efficiency and cost of renewable energy production must be maximized. Self-cleaning and anti-icing surfaces may be part of the solution as they could significantly increase the efficiency and reduce the maintenance costs of isolated solar cells and wind turbines.^7−9^

The investigation of superhydrophobic surfaces predominantly began with Barthlott and Neinhuis’s report on the lotus leaf in 1997.^10^ Since then, many have studied and fabricated surfaces that are water-repellent and often biomimetically inspired.^11−13^ As with superhydrophobic surfaces in nature, the properties of fabricated superhydrophobic surfaces are influenced by both their surface topography and surface free energy.^14^ An example of this is the lotus plant, which achieves superhydrophobic properties and self-cleaning properties by combing a low-energy waxy layer called the cuticle with a rough surface caused by topological microstructures.^15^

Both top-down and bottom-up strategies have been investigated when considering the fabrication techniques for superhydrophobic surfaces. Plasma etching,^16,17^ photolithography,^18,19^ and electron beam lithography^20,21^ are all excellent examples of top-down techniques that have been used to produce superhydrophobic surfaces. While these techniques have excellent tunability and accuracy, they are expensive and lack the scalability required when considering large surface areas.^22,23^ The use of these techniques to create templates and molds has also been explored, but there are still questions in regard to the scalability of these techniques and how durable the templates and molds would need to be so as to be cost-effective.^24,25^ Chemical vapor deposition (CVD) is a bottom-up technique that has been shown to produce viable superhydrophobic surfaces, but it again suffers from the same issues as top-down methods concerning costs and scalability.^26,27^ However, there are some bottom-up methodologies that have shown promise for economic viability.

Both self-assembly and the functionalization of nanoparticles have been used as the basis for bottom-up fabrication techniques.^28−30^ Traditionally, many of the superhydrophobic surfaces produced through bottom-up fabrication techniques have relied on the presence of fluorine to achieve low surface energy.^31^ These compounds have more recently been found to cause harm to the environment and human health, leading to industries looking to move away from composites containing fluorine.^32−34^ On the back of a report last year by Brunn et al.,^35^ a proposed restriction of around 10,000 per- and polyfluoroalkyl substances was put forward to the European Chemicals Agency by authorities in Denmark, Germany, the Netherlands, Norway, and Sweden.^36^ However, long-chain fatty acids have been identified by a number of reviews as having the potential to replace fluorinated compounds.^37−39^

As we look to move away from our reliance on fluorinated compounds for superhydrophobic properties, there are several approaches that can be considered. Agrawal et al.,^40^ Zhu et al.,^41^ and Heale et al.^42^ all fabricated superhydrophobic surfaces through a combination of nanoparticles and fatty acids, achieving x̅ WCAs of between 142 and 161°. Others combined their low-surface-energy compound with an additional base polymer in the hope of fabricating a more durable superhydrophobic surface. Zhi et al.^43^ explored both polyurethane and epoxy resin, with Li et al.^44^ also using epoxy resin, whereas Thasma Subramanian et al.^45^ used an acrylic resin. All three used spray application techniques to deposit their coatings on the desired surface, achieving WCAs of between 159 and 169°.

While there have been considerable developments regarding superhydrophobic surfaces over the past decade, there has been minimal commercial impact. Manoharan and Bhattacharya^39^ listed a number of limitations that may be holding the field back, including stability, durability, cost, health, environmental risks, etc. One way to remove some of these limitations is to attempt to functionalize a commercial product rather than starting from scratch. Jafari and Farzaneh^46^ took a novel approach and were able to modify a commercial latex with CaCO_3_ and stearic acid to achieve a WCA of ∼158° using spray deposition. Zhuang et al.^47^ produced robust superhydrophobic surfaces with a WCA of ∼160° and a sliding angle < 1° by functionalizing a commercial epoxy resin with polydimethylsiloxane through aerosol-assisted CVD.

While the combination of a latex polymer and a spray deposition technique is an easily scalable technique, latex polymers lack the durability of more robust polymers. By comparison, the combination of an epoxy resin polymer and a CVD technique produces a robust product, but CVD techniques lack the straightforward scalability of spray deposition techniques. This paper will look to advance on these concepts by outlining an approach capable of achieving an inexpensive, nontoxic, and accessible superhydrophobic surface using readily available commercial products. This will be done by first outlining a rapid method for the functionalization of nanoparticles with fatty acids before modifying a commercial polyurethane coating and a commercial epoxy resin with nontoxic fatty acids and nanoparticles. The coatings were deposited onto glass microscope slides using a spray technique before undergoing characterization and functional testing.

## 2 Experimental Section

### 2.1 Materials

A commercial polyurethane coating, a commercial epoxy resin, and Evonik Acematt HK 400 SiO_2_ particles (≥98%, 6.3 μm) were supplied by Alto Ltd. Stearic acid (C_18_H_36_O_2_), palmitic acid (C_16_H_32_O_2_), 100 nm zinc oxide (ZnO) nanoparticles (79.1–81.5%), acetone, and ethanol were all purchased from Sigma-Aldrich. The Kit 300B gravity-fed spray gun and air hose kits were purchased from Clarke International.

### 2.2 Fabrication of Hydrophobic Particles

A 1.5 g portion of fatty acid was dissolved in 50 mL of acetone at 50 °C while being stirred at 500 rpm and covered with a watch glass. The solution was then left for 20 min until the fatty acid was fully dissolved. Next, 1 g of particles (SiO_2_ or ZnO) were added to the solution and stirred with heating for 20 min. The particles were then washed three times and isolated using centrifugation at 4000 rpm for 5 min. The particles were then dried overnight in a vacuum oven at 50 °C. A mesh was then used to break up the solid into a powder.

### 2.3 Fabrication of Hydrophobic Coatings

0.25–3 g of stearic acid was dissolved in 50 mL of acetone at 50 °C while being stirred at 500 rpm and covered with a watch glass. The solution was then left for 20 min until the stearic acid was fully dissolved. Next, particles (SiO_2_ or ZnO) were added to the solution and stirred with heating for 20 min. The amount of particles used was dependent on the amount of fatty acid, with SiO_2_ used at a 1:6 *W*/*W* ratio to fatty acid, whereas ZnO was used at 2:3. 10 g of commercial polymer was mixed using 7 parts base and 3 parts hardener, which was then added to the suspension and stirred with heating for 20 min.

Immediately prior to application, the suspensions were sonicated and shaken for ∼5 min. For spray application, ∼30 mL of solution was added to a spray gun set at 3 bar using a 24 L air compressor. The suspension was then applied to the surface with the spray gun’s aperture opened by rotating the aperture control 0.5 rotations. Samples were then left for 48 h to dry on a bench at room temperature before the spray step, and the drying step was repeated to add a second coat.

For dip-coat application, a microscope slide was dipped into the uniform suspension for ∼2 s before being removed and left to dry for 48 h on a bench at room temperature. Once dry, the dip-coating process was repeated to add a second coat.

### 2.4 Material Characterization

Infrared transmission spectra were obtained by using a Brüker Alpha Fourier transform infrared (FT-IR) spectrometer with a platinum-attenuated total reflection attachment. All spectra were obtained from the accumulation of 16 scans per sample, under an analysis range of 400–4000 cm^–1^.

Topographic surface imaging was completed by using a JEOL JSM-6701F field emission scanning electron microscope. Samples were coated with gold prior to analysis and were analyzed with an acceleration voltage of 10 kV.

### 2.5 Functional Testing

WCA measurements, contact angle hysteresis (CAH) measurements, and rolling angle measurements were taken using a Krüss DSA25E droplet shape analyzer. For the WCA measurements, a DS3252 dosing unit was used to apply a 5 μL droplet to each coating at room temperature. A Young–Laplace fit was then used to calculate the contact angle of each droplet. Each measurement was performed in triplicate, with all reported measurements having a standard deviation of <0.5°.

For the CAH measurements, a protocol was adapted from Huhtamäki et al.^48^ A syringe dosing unit was used to dispense and aspirate a water droplet onto the surface of each coating. Over a minimum of 549 steps, the advancing and receding contact angles (RCAs) were measured. The CAH was then calculated by measuring the difference between the average advancing contact angle (ACA) and the average RCA.

For the rolling angle measurements, the tip of the syringe dosing unit was fixed 10 mm above the sample surface. The sample stage was then set at 1° intervals up to 10°, with a 13 μL water droplet dispensed onto the surface below between intervals. The rolling angle was defined as the angle at which the sample stage was set and at which point a water droplet dispensed would not stay on the surface. The experiment was repeated three times in a row to confirm the result.

Stain testing was performed by securing the sample at an 80° angle. Separate drops of 20 ppm of crystal violet, instant coffee, and wine were then applied to the surface using a Pasteur pipet.

A tape test was performed by firmly adhering Scotch Magic Tape to a surface before pulling it off in a single motion. This process was repeated nine times, using a fresh strip of tape each time, before any measurements were taken.

## 3 Results and Discussion

For the coatings, a number of variables were considered, each of which had an impact on the surfaces’ wetting properties or durability. Both ZnO and SiO_2_ particles were investigated as possible scaffolds, with both palmitic acid and stearic acid being investigated as functionalization agents. Palmitic acid and stearic acid were used due to their innate low surface energy, which is required if a surface is to repel water. Other variables that also impacted the surface properties were the fatty acid-to-polyurethane ratios and the spray distance. Formulations were produced with the minimal amount of solvent that would allow them to spray without clogging the spray gun.

Functionalized particles were produced by functionalizing 1 g of either ZnO or SiO_2_ particles in 1.5 g of fatty acid dissolved in acetone solution for 20 min. Centrifugation was then used to remove the functionalized particles from the solution before they were washed 3 times with acetone. Once dry, the functionalized particles were added to double-sided tape so their wetting properties could be analyzed. While these initial results showed that there was little difference in the wetting properties of particles functionalized with stearic acid as opposed to palmitic acid, they did show a significant difference when comparing the ZnO and SiO_2_ particles.

While prefunctionalized or pretreated SiO_2_ has previously been functionalized with fatty acids,^42^ attempts to functionalize pure SiO_2_ particles were unsuccessful. This is unsurprising when considering the proposed functionalization mechanism. As fatty acids are long-chain carboxylic acids, hydrogen can be removed from the terminal hydroxyl group when dissolved in acetone. It is this resulting polar end of the molecule that is then capable of reacting with the particle surfaces. There are a number of factors that may influence the interactions between the fatty acid and the SiO_2_ particles. First, SiO_2_ is less polar than ZnO, which could contribute to the reduced interaction between the dissolved fatty acid and the particles. There may also be a hydration layer on the surface of the particles interfering with the SiO_2_ and fatty acid interactions. Although this can be removed by treating the particles prior to functionalization, this would require an additional step. ZnO is more polar by nature and could easily be functionalized with the fatty acids in a relatively short period of time, resulting in an x̅ WCA of ∼173°.

**Figure 1:**
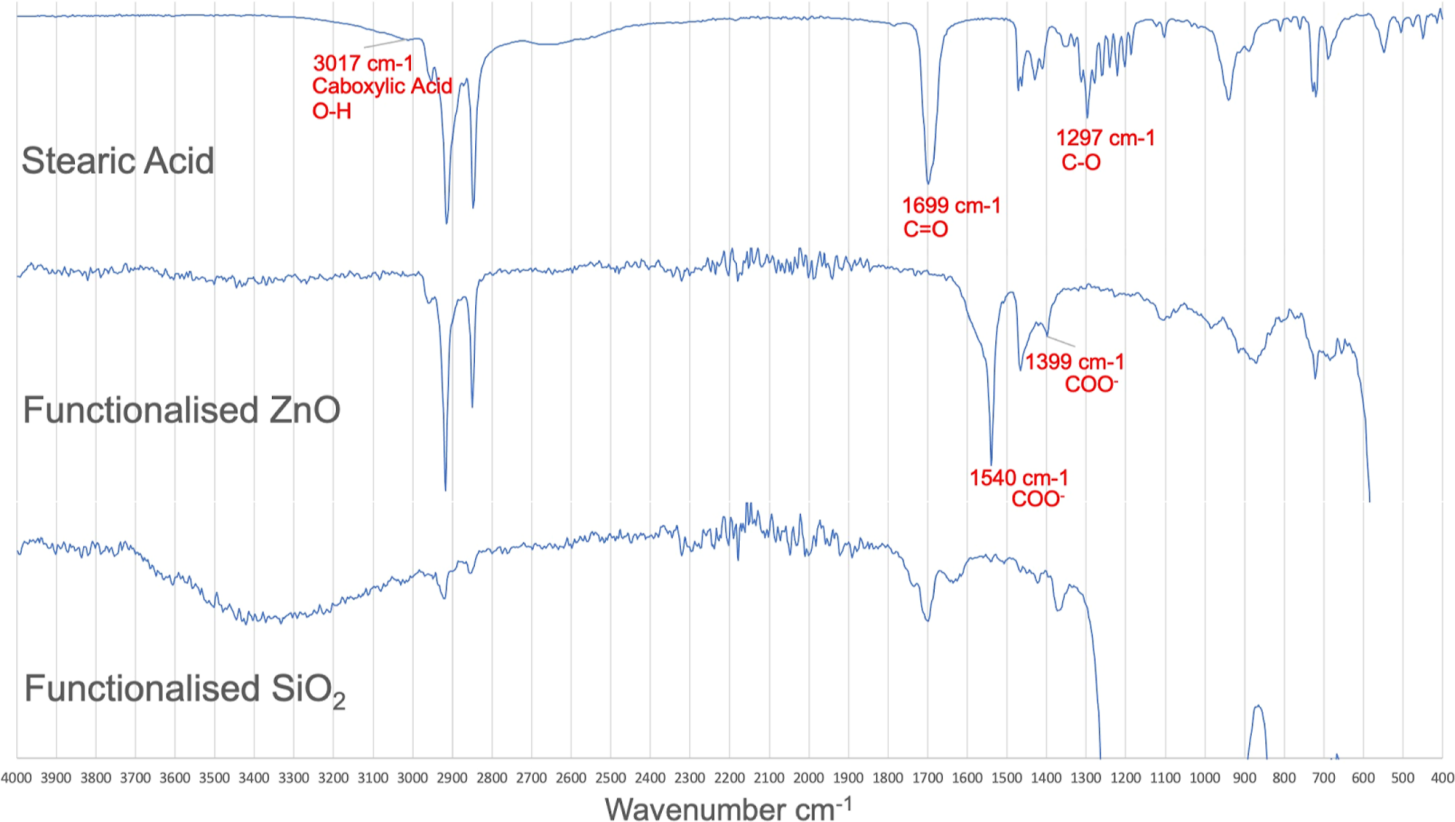
FT-IR analysis of stearic acid (top), functionalized ZnO (middle), and functionalized SiO_2_.

The functionalization of ZnO with the fatty acid and the nonfunctionalization of SiO_2_ were supported by FT-IR analysis (Figure 1). The fatty acids have a notable C=O peak at 1699 cm^–1^ and a large C–O peak at 1297 cm^–1^.^49,50^ These peaks are of particular note as once the fatty acid becomes bound to the surface through the proposed mechanism, the oxygen atoms go into resonance, eliminating the C=O and C–O peaks. These peaks are instead replaced with a symmetric COO^–^ stretch at 1399 cm^–1^ and an asymmetric COO^–^ stretch at 1540 cm^–1^ that occur due to the resonance.^51^ While these resonance peaks can be observed in the spectrum for the functionalized ZnO as expected, they were not observed in the postfunctionalization SiO_2_ FT-IR. In fact, the combination of the spectrum for the postfunctionalized SiO_2_ and their wetting properties suggests that the fatty acid was not bound to the particles and that all the fatty acid was removed during the wash step.

The next step was to formulate the mixture for coating. This was done by first dissolving the stearic acid in 50 mL of acetone using heat and stirring before adding the particles, followed by 10 g of premixed commercial polyurethane or epoxy resin polymer (3 part hardener and 7 part base). While the amounts of solvent and polymer were kept consistent, the amount of metal oxide particles and stearic acid was varied to allow for different component ratios. The solvent was then added to the suspension to give a final volume of 50 mL.

Coatings were then applied to a glass microscope slide using dip-coat or spray methods. Immediately prior to application, the suspensions were sufficiently shaken and sonicated to give a uniform suspension (∼5 min). The dip-coat application method simply required a microscope slide to be dipped into the uniform suspension before being removed ∼2 s later. The spray coat method used a compressed air spray gun set at 3 bar, with the aperture opened in the minimal amount that still allowed for the suspension to freely pass through and not clog the gun. Coated samples were then left to air-dry for 24 h before being tested.

**Figure 2:**
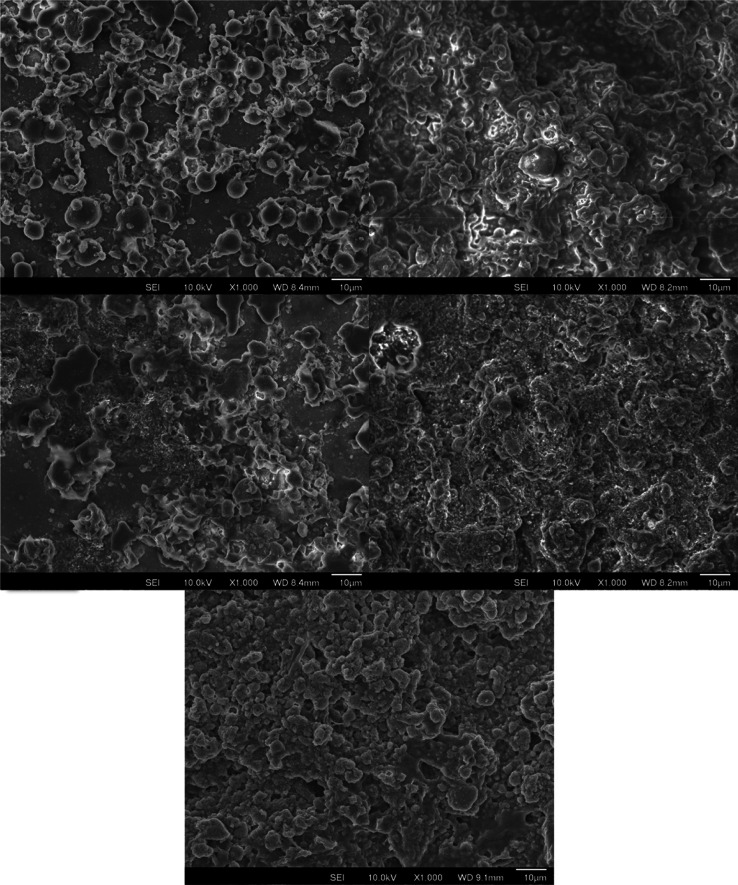
SEM images of polyurethane coating containing stearic acid, applied via dip coating (top left). Polyurethane coating containing stearic acid, applied via spray coating (top right). Polyurethane coating containing ZnO and stearic acid, applied via dip coating (middle left). Polyurethane coating containing ZnO and stearic acid, applied via spray coating (middle right). Polyurethane coating containing SiO_2_ and stearic acid, applied via spray coating (bottom).

Despite the use of sonication and shaking, the solvent and solid components of the suspension containing the epoxy resin remained firmly separated, with a uniform suspension being unachievable as the solid phase kept crashing out of the solvent. This meant that we could proceed with only the solution containing the polyurethane. Once the polyurethane coatings were dry, they were analyzed and compared. As can be seen in Figure 2, the spray coating method produced surfaces that were more consistently rough when compared to those of the dip coating methods. However, when investigating different spray distances, it was found that due to the volatility of the acetone and the inconsistent environmental conditions (the temperature of the lab could not be controlled and resulted in variations of the temperature by up to 20 °C), the ideal spray distance varied day to day. The impact of this was that if a surface was coated from too close a distance, the surface lacked roughness caused by the hierarchical buildup of the coating, resulting in surfaces with good durability but reduced WCAs. On the other hand, if the surface was coated from too far away, the acetone evaporated away from the suspension in the air, effectively meaning a dry powder was being deposited on the surface. This resulted in surfaces with good WCAs but poor durability. Overall, the ideal spray distance fell in the range of 30–60 cm from the surface.

When comparing coatings of different polyurethane to stearic acid ratios, a balance needs to be found between superhydrophobicity and durability. Coatings of different ratios were formulated by mixing 10 g of polyurethane with 0.25–3 g of stearic acid. Tape tests unsurprisingly showed that the 40:1 (polyurethane/stearic acid) coating was the most durable. This was expected as the polyurethane is harder than stearic acid; therefore, the greater the fraction of polyurethane, the greater the expected durability of the surface. This trend continued with testing, which also showed that the coating with a 10:3 ratio was the least durable, although any surface with a polyurethane content of less than 5:1 had poor durability. From investigations using 0.25 g fractions of stearic acid, it was discovered that the coatings reached their optimal hydrophobic properties at a 10:1 ratio. These surfaces also maintained reasonable durability, with them being resistant to tape testing and somewhat scratch-resistant. While surfaces with lower stearic acid content had improved scratch resistance, there was also a noticeable drop-off in WCA for each 0.25 g reduction of stearic acid.

FT-IR analysis of the coatings provided some intriguing and unexpected results. As could be seen when analyzing the ZnO particles functionalized with fatty acids, the binding between the ZnO and the fatty acid results in a resonance structure that produces a distinctive symmetric COO^–^ stretch at 1399 cm^–1^ and an asymmetric COO^–^ stretch at 1540 cm^–1^.^51^ However, when analyzing the coating’s spectra, neither of these peaks could be observed. As there is very little difference between the spectra of the pure polyurethane, the polyurethane mixed with stearic acid, and the polyurethane mixed with stearic acid and ZnO (Figure S1), it is possible that the peaks associated with the polyurethane were of such high intensity that they were masking other peaks.

**Figure 3:**
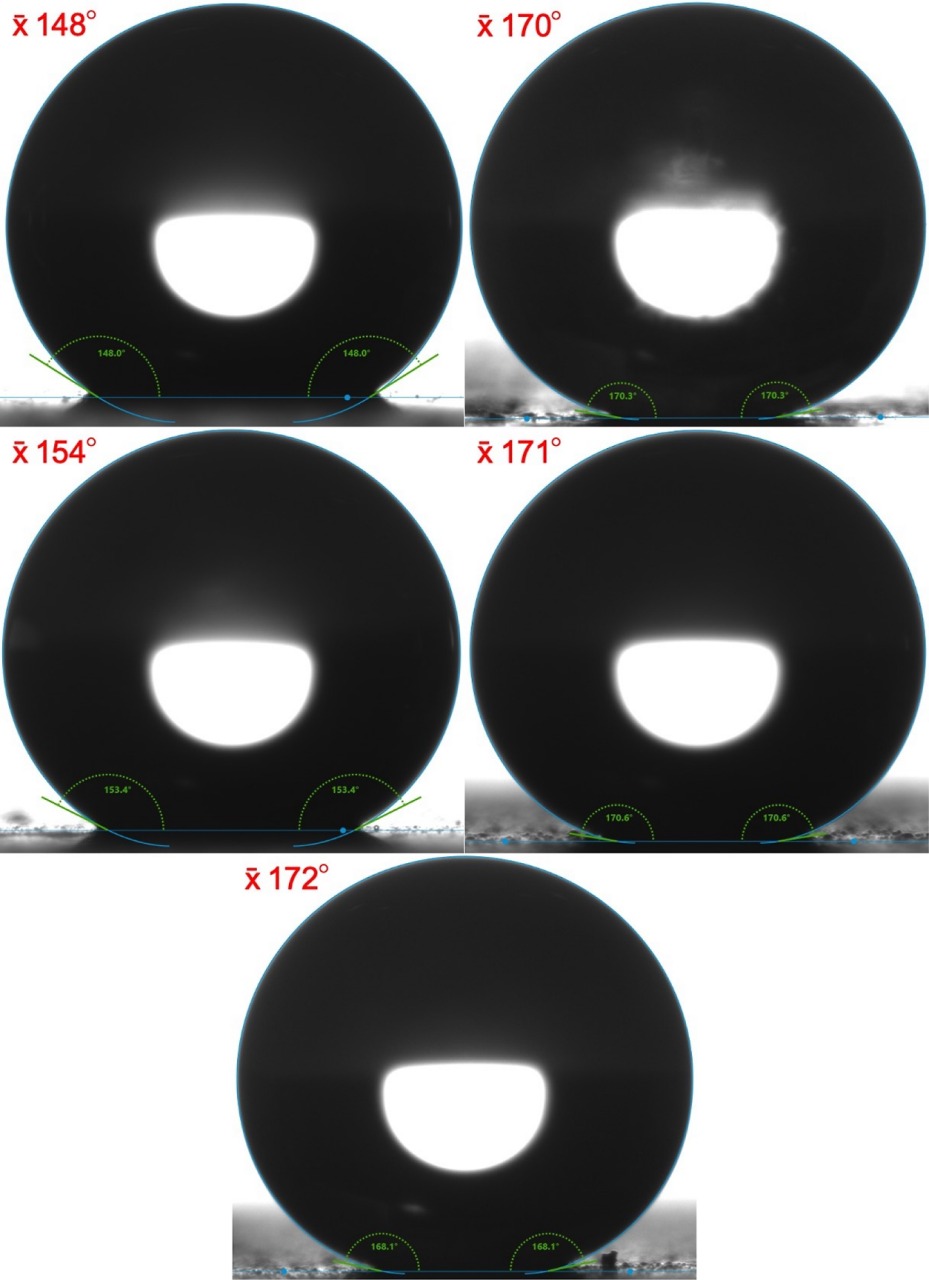
WCA of polyurethane coating containing stearic acid, applied via dip coating (top left). Polyurethane coating containing stearic acid, applied via spray coating (top right). Polyurethane coating containing ZnO and stearic acid, applied via dip coating (middle left). Polyurethane coating containing ZnO and stearic acid, applied via spray coating (middle right). Polyurethane coating containing SiO_2_ and stearic acid, applied via spray coating (bottom).

Functional testing was then carried out on coatings made up of 10 g of polyurethane, 1.5 g of stearic acid, and 1 g of ZnO particles or 0.25 g of SiO_2_ particles. Although 0.25 g of ZnO was sufficient, a higher concentration was used as there is no noticeable impact on the surfaces’ wetting properties, but it does have innate antimicrobial properties that SiO_2_ does not have. This may increase the surfaces’ viability for antibiofouling applications, but the testing of the surfaces’ antibiofouling properties is yet to be carried out. Standard drop shape analysis was used to show that while the pure polyurethane had hydrophobic properties (x̅ WCA of ∼95°), both the dip- and spray-coated surfaces had superhydrophobic properties. However, while the dip-coated surfaces’ x̅ WCAs did not exceed 154°, some of the spray-coated surfaces’ x̅ WCAs exceeded 170° (Figure 3). When analyzing both the IR analysis and the scanning electron microscopy (SEM) imaging, there was little difference between surface-containing particles and those without. This trend continued when looking at the x̅ WCAs of the sprayed surfaces, suggesting that the presence of the nanoparticles may not be required.

When the x̅ WCAs obtained were compared to those found in the literature, they were found to be very high. Looking across five reviews, most surfaces failed to achieve water x̅ WCAs ≥ 170°, with even fewer materials achieving a ≥170° x̅ WCA while not being reliant on a fluorinated compound.^52−56^ While Sharifi et al.^57^ were able to produce a fluorine-free superhydrophobic surface with a WCA ≥ 170°, their method required a TiO_2_ feedstock suspension to be suspension plasma sprayed onto a grit-blasted substrate. By comparison, our spray coating could be formulated from scratch in a one-pot method and sprayed onto a substrate in under an hour. When specifically comparing the surface to superhydrophobic polyurethane surfaces from some of the more recent publications, the surface had a greater x̅ WCA than that of the other reported surfaces.^58−60^ This was despite being fluorene, vastly reducing the environmental impact of the surface compared to that of the equivalent fluorene-containing compounds in the literature.

**Figure 4:**
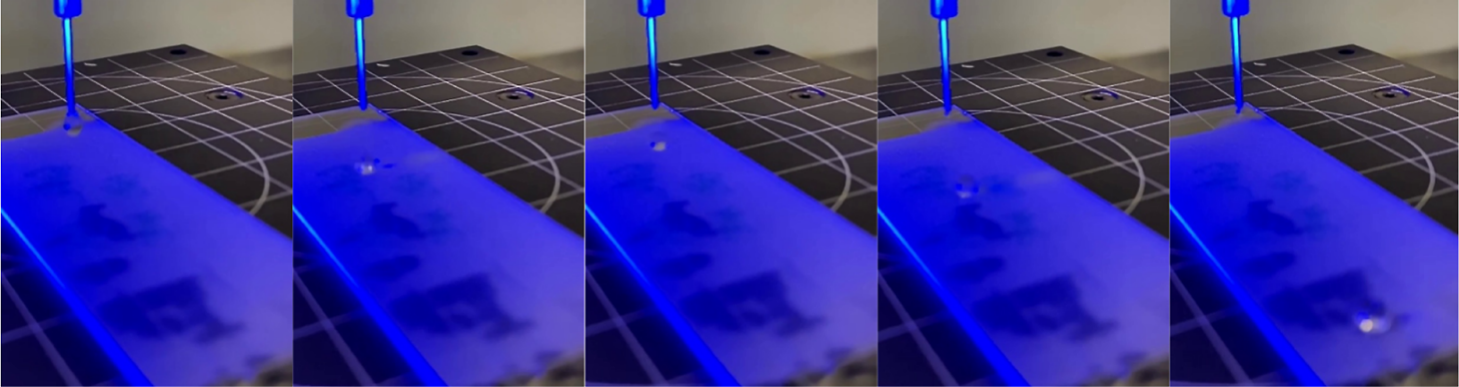
Demonstration of how a rolling angle measurement is taken. The stage is set to a designated angle, at which point a 13 μL water droplet is applied to the surface using a syringe dispenser raised 10 mm above the surface. If the water droplet was unable to stay on the surface, the current angle was deemed the surface’s rolling angle.

Another property to be determined was the coating’s surface rolling angle. However, there is very little consensus on how this measurement should be carried out or what qualifies as a measurement. For instance, neither Qi et al. nor Penna et al. reported the height at which their water droplet was dropped from or the volume of their droplet, both of which we found significantly impacted rolling angle measurements.^61,62^ Another issue is at what point should a measurement be taken or be valid. Is it when a droplet moves at all or when the droplet clears the surface? Due to these discrepancies, the rolling angle was clearly defined as the angle of tilt a surface needed to be at, to which a 13 μL water droplet dropped from 10 mm above the surface would be unable to stay on the surface it was applied to, with the implementation of this method being shown in Figure 4. Testing was used to determine that the rolling angle of the spray coatings containing no nanoparticles was 3° and that containing ZnO was 2°, while the rolling angle of the SiO_2_ surface was 1°. However, the rolling angle for the dip-coated surfaces was determined to be in excess of 10°, the maximum angle tested.

The final analysis of the surfaces’ wetting properties was an inspection of the surfaces’ CAH. As CAH is a measure of a surface’s ability to keep a droplet in place, it does not specifically determine if a surface is superhydrophobic.^63^ Surfaces with a low CAH value are better described as either superhydrophobic or have the potential to be superhydrophobic. This is because surfaces with low surface energy adhere poorly to water molecules, regardless of their surface roughness. Superhydrophobic surfaces are reliant on having both low surface energy and surface roughness. This means that if a surface has a low CAH value and a low WCA value, it must be relatively flat.^64^ However, if a surface of the same chemical composition is roughed, then it should see an increase in its WCA value.

In theory, the CAH is a surface’s ability to keep a droplet in place, and as such, the results should follow the same trend as the rolling angle analysis.^63^ The CAH was determined using a method by Huhtamäki et al.^48^ where the difference between the ACA and the RCA measurements is deemed to be the hysteresis. The ACA was measured as a water droplet was dispensed and expanded onto the surface being tested, with the RCA then measured as the droplet was being retracted off the surface. CAH measurements were taken for the two samples that had previously exhibited the best wetting properties, resulting in a CAH value of 2.05 for the coating containing ZnO and a value of 5.58 for the value containing SiO_2_.

These CAH results were slightly surprising as the initial expectation was that they would match the rolling angle results. While initially surprising, when the methodology of the CAH testing is considered, along with the two surfaces’ roughness (Figure 2), it is possible to form a hypothesis about what is happening. As CAH measures a surface’s ability to keep a droplet in place, with increasing values corresponding to increased adhesion, surfaces with a lower surface energy should have a low CAH value. Like the WCA and rolling angle measurements, good CAH values are reliant on low surface energy. However, while WCA and rolling angle analysis allow for the surface to simply repel the water, CAH analysis forces the water onto the surface. This means that unlike WCA and rolling angle measurements, which see a positive correlation between surface roughness and their superhydrophobic measurements, CAH measurements may decline when a surface becomes too rough. This added force may cause surface structures to penetrate the droplet’s surface, partially pinning it and reducing the water’s ability to freely move across the surface.

The next stage was to test the surfaces’ resistance to staining. Stain testing was performed by placing a sample at an 80° angle, then using a Pasteur pipet to apply a drop of a staining liquid to each surface. The stains used were crystal violet (20 ppm), red wine, and instant coffee. When the stain testing was carried out, the surfaces were able to completely repel all 3 liquids, resulting in no staining. Besides that, when samples were placed at a 10° angle and covered in loose dirt (glitter), the samples showed self-cleaning properties. This was done by simply rolling water droplets down the surface and through the dirt, which was removed by the rolling droplet.

Finally, the durability of the surfaces was tested by using a tape test. This test simply required the firm application of 10 strips of Scotch Magic Tape to the surfaces and their removal. After testing all the sprayed surfaces, all coatings maintained their wetting properties admirably with a ≤2° change in the x̅ WCA of the coating containing ZnO (171–169°), SiO_2_ (172–170°), or no nanoparticles (170–169°). The surfaces also appeared to be durable as little to none of the coating was removed by the Scotch Magic Tape.

## 4 Conclusions

This paper has shown a quick and simple route to an inexpensive, nontoxic, and accessible superhydrophobic surface. This was obtained by functionalizing a commercial polyurethane product with readily available fatty acids and ZnO nanoparticles using a spray technique. Surfaces were shown to have excellent superhydrophobic properties, with some x̅ WCAs exceeding 170°, as well as antistaining and self-cleaning properties. We have also explained the importance of using a polar nanoparticle when attempting to functionalize particles quickly and with minimal steps.

While we were able to functionalize a commercial polyurethane product, the same formulation was not compatible with a commercial epoxy resin product, despite evidence of epoxy resins being functionalized in the literature under similar conditions. The failure of the epoxy resin solution may be due to the specific formulation of the polymer or may be due to an additive in the commercial formula interfering with one of the reagents being used in the functionalization process. Despite this, this concept has been shown to be promising, yet more work is still required before the product can overcome all the limitations listed by Manoharan and Bhattacharya^39^ and prove to be commercially viable. If these limitations can be overcome, this concept could produce superhydrophobic surfaces rapidly through spray deposition at room temperature.

## Supporting Information Available

Optimized methodology for the fabrication of the hydrophobic coatings; FT-IR analysis of pure polyurethane, functionalized polyurethane with stearic acid, and functionalized polyurethane with stearic acid and ZnO; XPS survey of functionalized polyurethane with stearic acid and ZnO; and staining tests on the different coatings (PDF)
