# Supplementary material for: Modification of Commercial Polymer Coatings for Superhydrophobic Applications
Source: ACS Omega. 2024 Feb 2;9(6):7154–62. doi: 10.1021/acsomega.3c09123 (PMC10870754; doi:10.1021/acsomega.3c09123)
Supplement: Supplementary file 1 — ao3c09123_si_001.pdf [file ao3c09123_si_001.pdf]

Supporting Information

The modification of commercial polymer coatings for superhydrophobic applications

Sam S. Cassidy[1], Kristopher Page[1], Cesar III De Leon Reyes[1], Elaine Allan[2], Ivan P. Parkin[1], Claire J. Carmalt[1]*

*c.j.carmalt@ucl.ac.uk

1 Materials Chemistry Research Centre, Department of Chemistry, University College London, London, United Kingdom

2 Department of Microbial Diseases, UCL Eastman Dental Institute, Royal Free Campus, University College London, London, United Kingdom

Supporting Information

Optimised methodology for the fabrication of the hydrophobic coatings:
1.5 g of stearic acid was dissolved in 50 ml of acetone at 50 °C, while being stirred at 500 rpm, and covered with a watch glass. The solution was then left for 20 min, until the stearic acid was fully dissolved. Next particles either 1 g of ZnO or 0.25 g of SiO_2 were added to the solution and stirred with heating for 20 mins. 10 g of commercial polymer was mixed using 7 parts base and 3 parts hardener, which was then added to the suspension and stirred with heating for 20 mins.

Immediately prior to spraying, the suspensions were sonicated and shaken for ~5 min. ~30 ml of solution was then added to a spray gun set at 3 bar, using a 24 L air compressor. The suspension was then applied to the surface with the spray gun's aperture opened 0.5 rotations. Samples were then left for 48 h to dry on a bench at room temperature before the spray step and drying step was repeated to add a second coat.

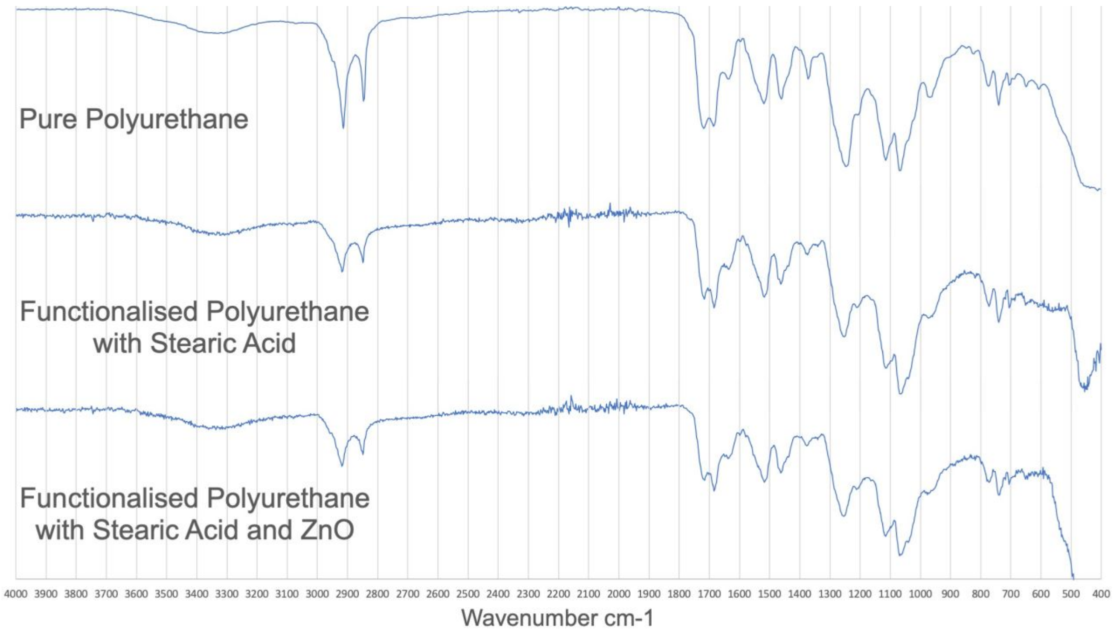

Sup Figure 1 FT-IR analysis of pure polyurethane (top), functionalised polyurethane with stearic acid (middle), functionalised polyurethane with stearic acid and ZnO.

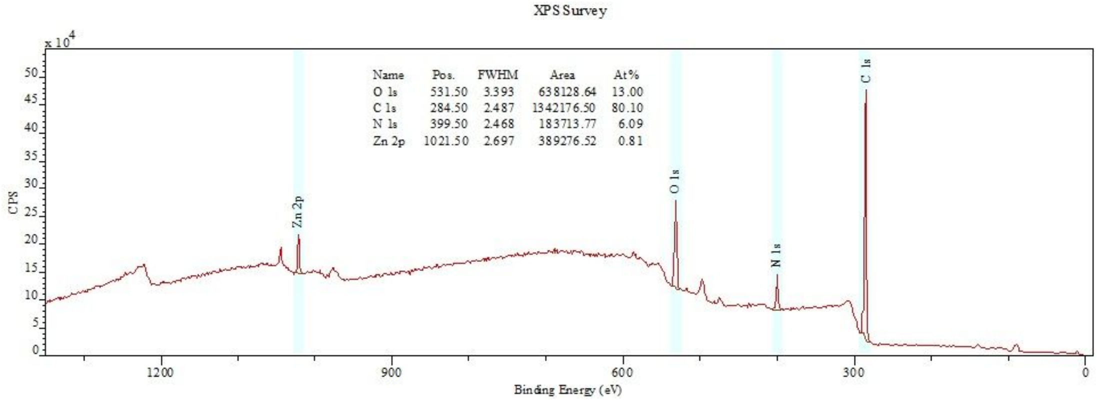

Sup Figure 2 XPS survey of functionalised polyurethane with stearic acid and ZnO

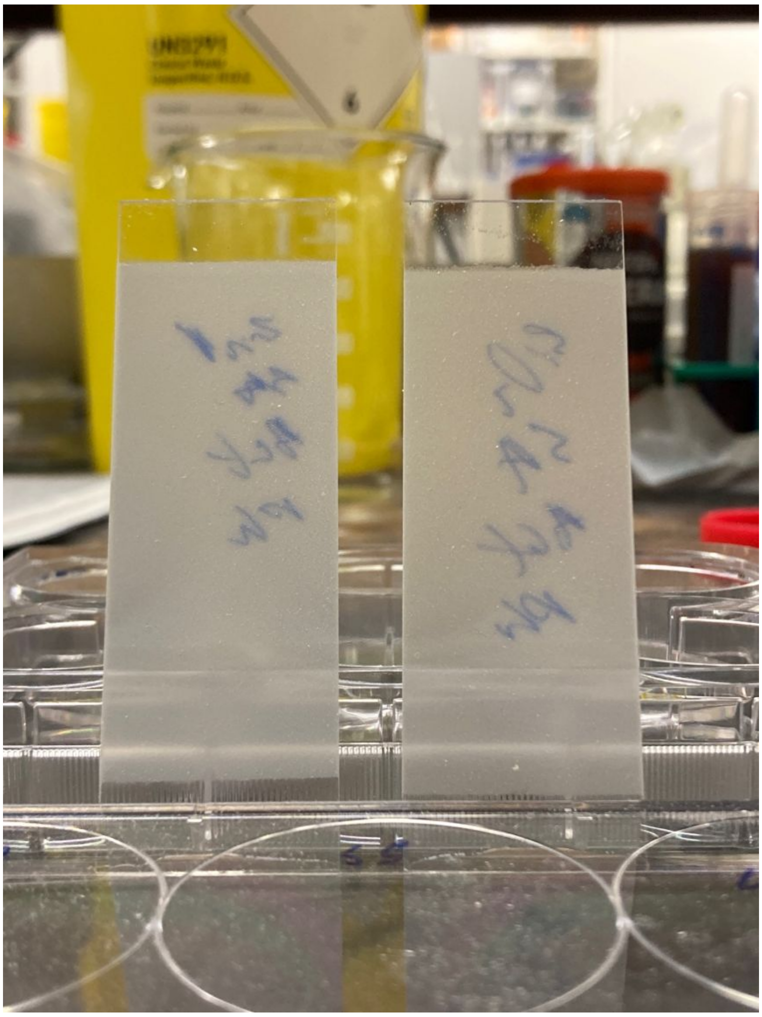

Sup Figure 3 Polyurethane coating containing ZnO and stearic acid (left). Polyurethane coating containing ZnO and stearic acid SiO_2 (right). Each sample was tested with 20 ppm crystal violet, instant coffee, and wine. As both surfaces were able to repel the solutions, they were left unstained
